# Facilitators and barriers to health professionals’ competence in delivering quality primary health care in the digital era in Amhara region, Ethiopia: an exploratory qualitative study

**DOI:** 10.1186/s12875-025-03003-9

**Published:** 2025-09-29

**Authors:** Gebeyehu Tsega, Mirkuzie Woldie, Gizachew Yismaw, Getu Degu

**Affiliations:** 1 Department of Health Systems Management and Health Economics, School of Public Health, College of Medicine and Health Sciences, Bahir Dar University, P.O. Box 79, Bahir Dar, Ethiopia; 2 Fenot Project, Department of Global Health and Population, Harvard T.H. Chan School of Public Health, Addis Ababa, Ethiopia; 3 Department of Health Policy and Management, Faculty of Public Health, Jimma University, Jimma, Ethiopia; 4 Health Research Development Directorate, Amhara Public Health Institute, Bahir Dar, Ethiopia; 5 Department of Epidemiology, School of Public Health, College of Medicine and Health Sciences, Bahir Dar University, P.O. Box 79, Bahir Dar, Ethiopia

**Keywords:** Pre-service education, Competence, Continuous professional development, Amhara region, Ethiopia

## Abstract

**Background:**

Despite the critical role of professional competence in providing quality primary health care, evidence on the facilitators and barriers to health professionals’ competence is limited. Therefore, we explored factors influencing health professionals’ competence in delivering quality primary health care in Amhara region, Ethiopia.

**Methods:**

A qualitative case study was conducted in Amhara region from November 1 to December 30/2023. We conducted key informant interviews with five faculty members from training institutions, five health service or facility managers, and five study participants from non-governmental organizations. Additionally, we conducted in-depth interviews with twelve health professionals. The number of participants was determined based on information saturation, and purposive heterogeneous sampling was used. A thematic analysis was conducted to analyze the data. The Standards for Reporting Qualitative Research (SRQR) were used to report findings.

**Results:**

Factors influencing the competence of health professionals in providing quality primary health care were divided into two main categories: facilitators and barriers. The presence of educational technologies, advanced instructional methods, educational design, the education-for-life model, universities, colleges, CPD centers, and NGOs working on health workforce capacity development in the region were facilitators of health professionals’ competence to provide quality primary health care. On the other hand, identified barriers include poorly prepared and demotivated students, the rapid expansion of universities and colleges with inadequate infrastructure, weak external quality assurance systems, poor collaboration between education and health systems, and the impacts of conflict and COVID-19.

**Conclusion:**

Although facilitators exist to enhance health professionals’ competence in the region, higher education institutions have not effectively produced the competent professionals necessary for quality primary health care. Factors such as poor-quality pre-service education, inconsistent and poorly implemented CPD initiatives, and limited NGO-funded training have all contributed to the subpar competence of health professionals. There is an urgent need for collaboration between the Ministry of Health and the Ministry of Education to plan, revise, and implement health professional training, improve the quality of pre-service education, and optimize in-service training and CPD efforts.

**Supplementary Information:**

The online version contains supplementary material available at 10.1186/s12875-025-03003-9.

## Introduction

The World Health Organization’s global competency framework for universal health coverage (UHC) defines health professionals’ competence as their ability to provide quality healthcare that is effective, safe, people-centered, timely, equitable, integrated, and efficient. Competent health professionals are crucial for improving population health, enhancing health systems’ resilience, and fostering economic growth. Without them, achieving the highest attainable standard of health is impossible [1–3].

Hence, the effectiveness of health systems and the quality of care people receive depend on the competence of health professionals [4, 5]. In the era of disease outbreaks, conflict, and climate change, global and national political leaders prioritize the competence of health professionals as a top policy issue [1, 4, 6–11].

However, the literature indicates a shortage of competent health professionals, particularly in rural, remote, and underserved areas where primary health care units are often the main providers of health services [12, 13]. In low-income countries, chronic under-investment in health professional education and training, along with mismatched education and health system demands, exacerbates the shortage of competent health professionals [4, 14, 15]. Additionally, challenges in retaining competent health professionals include a lack of incentives, impractical policies, poor work environments, weak leadership, and unfavorable political and social contexts [12, 13, 16]. According to Johnson and colleagues’ framework, pre-service education-related factors influence the competence of health professionals. Health systems and regulatory factors also play a role [17].

Incompetent health professionals cause suboptimal care, leading to death, disability, economic burden, loss of trust between providers and clients, and staff dissatisfaction [12, 13, 18–20]. Each year, suboptimal care causes 8 million deaths in low- and middle-income countries, accounting for 60% of preventable deaths and loss of trust in health systems among over three-quarters of the population [12, 13, 20].

A whole-of-society (primary health care) approach in education and health systems can address health professionals’ competence challenges. The UN meetings in 2019 and 2023 and the Alma-Ata and Astana declarations highlight the need for competent health professionals to provide quality primary health care [1, 5, 21–23]. Strong accreditation, regulation, and collaboration between education and health systems ensure competent healthcare professionals [24]. The education-for-life model, with its three dimensions—lifelong learning, promoting and restoring health, and learning to live one’s life—supports continuous improvement in competence [19].

In Ethiopia, despite various attempts like continuous professional development (CPD), licensure exams, accreditation, quality assurance, and community-led evaluations, the low competence of health professionals at primary health care units often results in suboptimal primary health care [18]. A recent survey revealed that less than half (45%) of respondents received quality primary health care [13]. However, there is limited evidence on factors influencing the competence of health professionals in providing quality primary health care in Ethiopia in general and in the Amhara region in particular. Therefore, this study aimed to explore factors influencing the competence of health professionals in delivering quality primary health care in Amhara region, Ethiopia.

### Theoretical perspectives

Since one framework cannot adequately explain health professionals’ competence in delivering quality primary health care, we adapted the WHO global competency framework [1] and Johnson and colleagues’ framework [17]. Neither of these frameworks can solely explore the domains of quality in primary health care (PHC) and the factors influencing competence comprehensively. The first framework elucidates the domains of quality in primary health care, while the latter explains the factors that influence health professionals’ competence in providing quality primary health care.

### Quality primary health care

The WHO’s global competency framework for quality primary health care includes six domains: people-centeredness, decision-making, communication, collaboration, evidence-informed practice, and personal conduct. People-centeredness places people at the heart of all practices and empowers individuals and communities to take charge of their health. Decision-making involves interpreting evidence and making judgments based on relevance, timeliness, resources, and others’ needs. Communication is essential for guiding, informing, supporting, and collaborating through interactions and documentation. Collaboration requires cooperation with health professionals, caregivers, families, and the population. Evidence-informed practice integrates evidence, experience, and values to improve care quality, safety, and outcomes. Personal conduct, guided by ethical principles, impacts safety, quality, and trust in health care. This global competency framework was recently developed with a deliberate focus on delivering quality primary health care, following the Astana Declaration on PHC [1]. Researchers applied this framework in a previous study to understand the scope of practice of physician assistants and comparable professions in 2023 [25]. Hence, we used this framework in this study since it best suited for framing quality PHC.

### Johnson and colleagues’ framework

Johnson and colleagues’ framework emphasizes the various pre-service education factors that influence the competence of health professionals in delivering quality primary health care. This framework organizes factors related to students, curriculum, teachers and preceptors, infrastructure and management, clinical/community practice sites, and the health system. Students’ transition into professional practice depends on their academic performance, motivation, and access to resources. Instructor and preceptor expertise, combined with mentorship, bridge theoretical knowledge with practical application, fostering critical thinking. Institutional infrastructure—encompassing libraries, laboratories, and clinical training sites—supports learning by providing essential exposure to healthcare. A well-structured curriculum aligned with primary health care needs equips students with practical skills for real-world challenges. Clinical rotations and internships enhance competence through hands-on experience in diagnosis, treatment, and patient management. Beyond institutions, health system policies shape the effectiveness of training, ensuring that curricula uphold patient safety, ethical standards, and service delivery expectations [17]. Hence, this framework is best suited for exploring factors that influence the competence of health professionals in delivering quality primary health care.

## Methods

The Standards for Reporting Qualitative Research (SRQR) informed the process and guided the reporting of findings in this qualitative study. These standards enhance the transparency of all aspects of qualitative research by providing clear guidelines for reporting [26].

### Research paradigm and study design

This study was grounded in a constructivist philosophical framework. This philosophical foundation is commonly linked with qualitative methods and informal rhetoric. With this in mind, a qualitative case study, guided by a conceptual framework adapted from Johnson et al., [17] was conducted from November 1 to December 30, 2023. This conceptual framework outlines broad categories of factors related to students, curriculum, teachers and preceptors, infrastructure and management, clinical and community practice sites, and health system factors, including deployment practices and regulations, that impact the competence of health professionals. Hence, this framework was the best fit for the study. A qualitative case study was selected to facilitate the exploration of a phenomenon (in this case, factors influencing health professionals’ competence) within a specific context. A qualitative case study approach involves gathering data from multiple sources and considering different perspectives [27]. In this approach, researchers heavily consider participants’ viewpoints and subjectively interpret the phenomena. Constructivist research unfolds from individual perspectives, gradually revealing broad patterns and leading to comprehensive understandings [28].

### Study setting

The study was conducted in selected primary health care units, public universities/colleges, and NGOs within the Amhara region, with a special emphasis on Primary Health Care (PHC). Recognizing that PHC is the cornerstone for achieving Universal Health Coverage (UHC) and provides 90% of essential services [29], we specifically focused on the PHC level within the Amhara region.

Amhara region is one of Ethiopia’s most populous regional states and is also affected by conflict. It comprises 14 zones, 8 city administrations, and 180 districts (139 rural and 41 urban districts). According to the Ethiopian Central Statistics Agency, the region’s projected population is 30.9 million, with approximately 80% residing in rural areas [30]. Ethiopia’s economy, including the Amhara region, grew by 7.2% in 2022/23, but it remains one of the poorest countries, with a per capita GNI of $1,020. Youth unemployment is high at 25%, especially among females (30%), due to competence gaps and limited job opportunities [31]. Health expenditure per capita increased to $36.40 in 2019/20, still below the WHO’s recommended $86 [32]. Amhara region has 100 hospitals, 917 health centers, and 3,725 health posts. The region is supported by a total of 32,589 health professionals, of whom 40% are female. These professionals include 36.8% nurses, 13.4% midwives, 10.4% health officers, and 4% doctors [33].

### Study participants

Twenty-seven study participants were included based on information saturation. Information saturation represents a point of information redundancy or diminishing returns, indicating the juncture at which sufficient information power is attained [34]. Accordingly, we interviewed five faculty members from training institutions, five health service/facility managers, twelve health professionals, and five NGO participants.

### Sampling methods

Academicians, health service managers, health professionals, and NGO participants were selected through heterogeneous purposive sampling to gain diverse perspectives on the competence of health professionals [35].

### Data collection

Researchers and two experienced data collectors collected data from November 1 to December 30, 2023. The data were collected through various data collection methods, including key informant interviews (KIIs) and in-depth interviews (IDIs), using semi-structured interview guides. These semi-structured interviews focused on exploring stakeholders’ perceptions of several key areas related to factors influencing the competence of health professionals in Amhara Region, Ethiopia. The interview guides were developed based on previous literature [1, 19, 23] and included key and probing questions (Additional file 1).

Data were collected face-to-face: KIIs with academia, managers, and NGOs; IDIs with health professionals. In-depth interviews explored the lived experiences of health professionals regarding the barriers and facilitators to their competence in providing quality primary health care. Key informant interviews were conducted with individuals who possess extensive knowledge and expertise in the competence of health professionals to gain detailed insights and understand the context and nuances that influence competence. Suitable locations and languages were chosen for data collection: English for both KIIs and IDIs. The average interview duration was 50 min for both KIIs and IDIs. Audio records and notes were used during data collection.

### Data quality assurance

Data quality was assured based on Guba and Lincoln’s trustworthiness criteria (credibility, transferability, dependability, and confirmability) [36]. Techniques such as prolonged engagement, triangulation, and member checking were used to ensure data credibility. Transferability was assured by thick description and heterogonous purposive sampling. Dependability was ensured through a code book, audit trail, peer debriefing, and negative case analysis. Reflexivity, an audit trail, and peer debriefing assured confirmability. Audio-taped data transcriptions and translations were made; the accuracy of the transcripts was continuously cross-checked against the audio recordings. Additionally, data collectors were trained, and the study instruments/tools were pre-tested.

### Data processing and analysis

Verbatim audio recordings and non-verbal cues noted in the notebook were transcribed, followed by conceptual data translation. The transcribed and translated data were then analyzed using ATLAS.ti.9, a qualitative data analysis software. Content analysis, a qualitative research method, was employed to interpret and understand the data’s meanings, themes, and patterns. This process involved several key steps: selecting relevant content, coding the data into manageable categories, analyzing the coded data to uncover deeper meanings, and presenting the findings. After transcription and translation, the coded data were analyzed using thematic analysis. This process involved both framework analysis, which draws from the theoretical framework, and inductive methods, which emerge directly from the data. By combining these approaches, the study provided a comprehensive understanding of the themes and patterns within the data [37].

## Results

### Background of interviewees

The interviewees included twelve health professionals from health centers and primary hospitals, five from NGOs, five academics from universities and colleges, and five health leaders from the health system. The ages of the interviewees ranged from 25 to 45, and their educational statuses varied from diploma holders to PhD holders.

### Understanding of competence among interviewees

The understanding of health professional competence varies among health professionals, managers, academics, and NGO interviewees. Health professionals understood competence as the capability of delivering timely and safe patient care with less emphasis on public health practices. In contrast, health managers understood competence as the capacity to provide the maximum possible health services to individuals and populations at the lowest cost and in the shortest time. Academics and NGO interviewees generally viewed competence as possessing at least minimum proficiency in providing comprehensive and quality health services, which include disease prevention, health protection, and promotion, rather than solely focusing on treating sick individuals. Most interviewees agreed that health professionals should possess competencies in people-centeredness, communication, evidence-informed decision-making, collaboration, evidence-based practice, and personal conduct to provide quality primary health care.

### Understanding of quality primary health care among interviewees

Interviews with healthcare professionals, academics, managers, and NGO interviewees highlight a multifaceted understanding of quality primary health care (PHC). The interviewees identified three key areas of PHC: preventive, curative, and integrated services. Healthcare professionals emphasized the curative aspect of PHC, viewing quality PHC as safe and timely curative services. Managers focused on the preventive dimension, considering that quality PHC efficiently prevents diseases and promotes health. Academics and NGO interviewees highlighted both preventive and curative services, stressing the importance of public health practices and primary care for addressing individual and population health needs. Some interviewees viewed quality PHC as providing adequate care for those unable to afford specialized care, emphasizing the need for accessible and equitable health services.

Different perspectives on the importance of people-centered health services in all key areas (preventive, curative, and integrated services) were reported. While most interviewees stressed its significance, a few argued against its inclusion as a quality dimension due to Ethiopia’s low health literacy among people and shortage of health professionals. Essential elements for quality PHC, as identified by the interviewees, include effective communication, evidence-informed decision-making, teamwork and collaboration, evidence-based practice, and high-standard personal conduct. Interviewees reported that different factors related to pre-service education and the health system play a crucial role in shaping the competence of health professionals, thereby affecting the provision of quality PHC.

### Facilitators to health professionals’ competence in delivering quality primary health care

Interviewees identified various facilitators to health professionals’ competence during their education and within the health system. These facilitators were divided into subthemes: students, infrastructure and management, clinical and community practical sites, curriculum, instructors and preceptors, and factors related to the health system.

#### Facilitators related to students

Interviewees identified several facilitators related to students that enhance health professionals’ competence. According to their views, these facilitators include a substantial value for health professions, relatively high cutoff points for entering health fields, and new initiatives in pre-higher education. For example, most interviewees noted that many students have a positive attitude and high regard for health professions, especially medical doctors. Additionally, strategies to prevent cheating and grade inflation were mentioned, such as administering university entrance exams at universities instead of students’ schools, thereby moving away from practices prone to cheating and grade inflation. Some interviewees also reported that making universities autonomous could provide opportunities to set entry criteria for their prospective students in health fields, including their entrance exams before enrollment.

*[…] If you ask primary school students about their envisioned future profession*,* many will answer ‘medical doctor*,*’ reflecting their positive value for the health profession […] (KII-15).*

#### Facilitators related to infrastructure and management

Interviewees identified several facilitators that enable the production of competent health professionals to deliver quality primary health care (PHC) services in this subtheme. These facilitators included IT-facilitated education (such as augmented realities, virtual patients, and mixed realities), licensure/exit exams, initiatives to improve the quality of pre-higher education, graduate admission tests, presence of professional councils and associations, health science education centers at universities, competency-based education, competency frameworks, and accreditation. Additionally, the presence of national and subnational strategic plans for health professionals’ competency assessment, with a responsible structure for implementation, facilitates quality pre-service education to produce competent health professionals.

The interviewees reported that educational technology has been a game-changer in the health professionals’ education. It has made learning more accessible and flexible, allowing students to learn at their own pace and schedule, including using Free Online Learning Resources. With the help of educational technology, students can learn from anywhere in the world, with or without facilitators. Blended learning, such as flipped classrooms and virtual patients for clinical practices, is possible in this digital era. Moreover, interviewees pointed out machine learning and artificial intelligence can work as humans in educational activities that significantly improve the quality of pre-service education:

*[…] When utilized wisely*,* technology has the potential to make what was deemed impossible possible; for example*,* the internet has made it possible to connect with people from all over the world and access vast amounts of knowledge with just a few clicks[…] (KII-2).*

Most interviewees pointed out that different attempts have been made in Ethiopia, including in Amhara region, to improve the quality of education at all levels. These include initiatives such as licensure exam/COC, accreditation, the presence of a responsible body for external quality assurance such as the Ethiopian Education and Training Authority (ETA), the presence of NGOs (e.g., Jhpiego) working on improving pre-service education aligned with the national and subnational priorities:

*[…] Licensure exam has spillover effects on the quality of pre-service education in higher education institutions*,* makes them [universities and colleges] work their best to increase the pass rate of their students to be competitive and attract more students with high academic performance*,* alongside protecting the public from incompetent professionals […] (KII-5).*

#### Facilitators related to clinical and community practice sites

A few facilitators were identified in this subtheme: universities beginning to establish their teaching hospitals, the presence of research field sites, and community-based education sites (e.g., for team training and community training programs) at several universities:

*[…] When attending my BSc degree*,* I benefited from the community-based and team training programs*,* which provided opportunities to apply the knowledge gained from classroom lectures at community-based education sites […] (IDI-11).*

#### Facilitators related to the curriculum

In this subtheme, interviewees identified several curriculum-related facilitators. These included the presence of health professionals’ education experts with specialized training in medical education and curriculum design and the availability of advanced global and specific competency frameworks for health education programs. Additionally, introducing competency-based education in some universities facilitated health professionals’ competence. Some interviewees noted efforts to develop standard competency frameworks and entrustable professional activities (EPAs) for certain health professionals as facilitators:

*[…] Competency-based education can equip health professionals with the required competencies to address health needs and priorities […] (KII-11).*

#### Facilitators related to teachers and preceptors

Interviewees identified some facilitators for teachers and preceptors, including higher diploma education and training in pedagogy and andragogy for health instructors and preceptors, often in collaboration with NGOs. Additionally, capacity building of teachers through master’s and PhD programs was also recognized as a facilitator of health professionals’ competence:

*[…] Higher diploma education and training on effective teaching for health instructors can fill their teaching approach gaps […] (KII-8).*

#### Facilitators related to the health system

Most interviewees reported a range of facilitators for CPD and IST. These include the fact that CPD has become a national and subnational policy priority, the presence of a national CPD roadmap, the promotion of linking CPD with license, the presence of NGOs, universities, colleges, and CPD centers working on capacity building of health workforce in the region; the presence of IT facilitated e-CPD, the presence of the regulatory body and the promotion of a primary healthcare-oriented health system:

*[…] In the near future [in Amhara region]*,* CPD will be required for health professionals to re-license for health care. Educational technology provides an opportunity to continuously improve health professionals’ competence everywhere*,* with or without facilitators*,* including online courses* […] *(KII-4).*

### Barriers to health professionals’ competence in delivering quality primary health care

Several barriers hindering health professionals’ competence were identified, ranging from pre-service education to the health system. These barriers were categorized into subthemes: students, infrastructure and management, clinical and community practical sites, curriculum, instructors and preceptors, and health system factors. This categorization follows Johnson and colleagues’ framework, which served as the theoretical basis for this qualitative study.

#### Barriers related to students

In this subtheme, problems related to admission criteria, lack of motivation, negligence among students, IT-facilitated cheating, and credentialism (a tendency to get a certificate of degree as a ticket for employment rather than developing the requisite competencies) were reported as barriers to producing competent graduates:

*[…] The 12th-grade national exam score is the sole criterion for admission to health fields in most Ethiopian universities and colleges. While necessary*,* it is insufficient to ensure students are well-suited for these programs […] (IDI-12).*

Most interviewees reported that the poor quality of education in primary and secondary schools has resulted in poorly prepared and demotivated students. These ill-prepared students enter universities and colleges, and after graduation, some become instructors in higher education institutions while others join the healthcare system as practitioners. This situation affects the competence of health providers and instructors as they deliver care and teach students, creating a vicious cycle that perpetuates incompetence in the health and education systems:

*[…] Currently*,* poorly prepared and demotivated students are joining health education programs due to problems in the entry criteria […] (KII-4).*

Interviewees also pointed out that some students may use forged evidence to join higher education institutions, especially private colleges. Moreover, interviewees highlighted that illegal and unethical practices in Ethiopia’s education system have led to incompetent graduates, especially in private colleges:

*[…] Commercialization of health professionals’ education has become normal in our country so that private colleges*,* sometimes public*,* recruit students with forged certificates of grade 12 entrance exam results and provide degrees for them [students] without attending the educational programs […] (KII-2).*

#### Barriers related to infrastructure and management

In this subtheme, limited capacities in infrastructure and management (e.g., classrooms, library, simulation, and skill laboratories), weak regulations and governance, limited adaptive capacities for disasters like COVID-19, and conflict in higher education institutions were reported as barriers to producing competent graduates. For example, respondents from training institutions in Amhara region have indicated that it is common to commence educational activities before the completion of building construction. Furthermore, disruptions in both virtual and in-person classes have been frequently reported:

*[…] Universities and colleges have been graduating many students*,* beyond their capacity*,* without the required competencies*,* who are unable to provide quality care […] (IDI-10).*

Most respondents pointed out weak coordination (sometimes conflict) between the education and health system, limited national and international partnerships, and political interference:

*[…] Both of them [academics in health and health system] have the same goal of improving and protecting the health of the population and patients. Still*,* they work separately in a fragmented manner […] (KII-1)*.

#### Barriers related to clinical and community practice sites

In this subtheme, the lack of higher education institutions’ teaching hospitals, coordination between education and health system actors, political unrest and insecurity to go out for community-oriented practice, and inadequate clinical practice sites were reported as barriers to producing competent graduates. Most respondents also reported that health graduates lack practical experience due to limited opportunities for clinical and community-based practical experience, partly due to large class sizes, the absence of teaching hospitals in universities, and the lack of pedagogical and clinical competence of preceptors:

*[…] The flooding strategy at the expense of quality makes the clinical practice sites and simulation laboratories insufficient to accommodate many health students at a time*,* and insecurity due to conflict impedes community-oriented education such as team training programs (TTP)*,* community-based training programs (CBTP) and research fieldwork […] (KII-3).*

#### Barriers related to the curriculum

Outdated, fragmented, non-competency based, implausible, and unstandardized curricula, mismatch of competencies and health care practice, absence of nationally agreed competency framework and entrustable professional activities (EPAs), limited emphasis for interprofessional education, lack of need assessment while designing curriculum and absence of legal framework to implement and harmonize curricula across all higher education institutions were reported as curriculum-related barriers. Additionally, the interviewees noted that the curricula do not consider Ethiopia’s indigenous knowledge and wisdom. Most interviewees pointed out that the curricula (often not tailored to the actual health needs of individuals and populations in the country) focus more on theoretical knowledge at the expense of practical sessions, resulting in a mismatch between health professionals’ education and health care practice:

[…] *Health professionals’ education and healthcare practice are misaligned due to outdated*,* fragmented*,* and un-tailored curricula*,* resulting in shame and harm to clients among health professionals in their workplace […] (IDI-5).*

Most interviewees pointed out that health care is a teamwork that requires inter-professional education and collaborative practice. However, most health professionals’ education programs lack the platform to facilitate inter-professional education. Only limited inter-professional education strategies, such as team training programs, have been implemented in universities, but not in private colleges and those run by regional governments. Despite the growing international call for integrated people-centered primary health care, almost all key informants indicated that specialized care -biomedical model-oriented curriculum is prioritized over primary health care in higher education institutions. Some interviewees noted that primary care and essential public health functions are being overlooked in the curricula of educational programs:

*[…] Higher education institutions’ graduates are often ill-equipped to provide inter-professional collaborative and integrated people-centered primary health care services due to the design of educational programs around illness/diseases with siloed health professionals’ education […] (KII-5).*

#### Barriers related to instructors and preceptors

Incompetent (pedagogically and in subject matter) and demotivated instructors and preceptors, dual practice (moonlighting) and lack of compliance for implementing the curricula among instructors, lack of partnership with national and international faculty members, lack of capacity building for instructors were reported as barriers related to instructors and preceptors:

*[…] The instructors and preceptors are incompetent in pedagogy and subject matter knowledge*,* and most of them [instructors] lack clinical experience. They [instructors] use outdated PowerPoint and educational materials every year. They [instructors] are busy with non-academic work […] (IDI-9).*

#### Barriers related to the health system

Interviewees also identified several barriers rooted in system design and contextual issues within the health system. Factors negatively impacting in-service training and continuous professional development (CPD) included the lack of health professional regulation and accountability, absence of competency-based performance management, inadequate CPD implementation due to budget constraints and lack of operational and monitoring frameworks, failure to link CPD with re-licensure, and fragmented, donor-driven in-service training and political interference.

All interviewees reported that it is a common practice for junior and less competent health professionals to be assigned to rural health centers and primary hospitals. It was stated that primary health care facilities are “incompetent systems” (with mistakes and unfair/discriminated health care favoring political leaders and personally connected individuals). As a result, they provide incompetent care (provided by incompetent providers without clear explanation of cases for the patients). Unfortunately, after the junior health professionals become more competent (through experience and training), they are often transferred to general and comprehensive specialized hospitals in urban areas for better living standards. The interviewees suggested that this practice should be reconsidered, as the disease burden is high in remote and rural areas.

There were also reported problems of using available competent professionals with required competencies and assigning them to roles that do not match their field of study in the health system, especially at primary health care facilities. This is rooted in the use of “form 15” (an outdated and non-flexible regional health professional recruitment and development directive) and the pool system with no understanding of the unique context of the health system. This demotivates health professionals since it hinders personal and professional development:

*[…] Form 15 is not fit for purpose health workforce policy that was developed a long time ago*,* which may have worked better during that time*,* but now it does not work and does not accommodate the current health system demands and population needs. There is an occasion that approved positions filled by diploma holders with degree holders available in the market due to [use of] form 15[…] (IDI-4).*

The interviewees also pointed out that lack of understanding among government sectors and lack of good governance in each industry are barriers to health professionals’ competence:

*[…] The pool system*,* which lacks a deep understanding of the health system’s unique features*,* is vulnerable to corruption and bias during deployment and transfer of the health workforce; assignment and transfer are not based on merit [competence] but rather on personal connections*,* political affiliations*,* and nepotism that lead to demoralizing the health professionals […] (IDI-8).*

Most interviewees indicated that health professionals encounter significant obstacles in delivering quality PHC, particularly in primary healthcare settings. These challenges encompass insecurity, scarce resources, inadequate infrastructure, including internet and mobile networks, clean water and housing deficiencies, the lack of libraries and continuous professional development (CPD) opportunities, low salaries and benefits, and more:

*[…] Providing high-quality integrated people-centered services becomes an illusion due to limited resources*,* poor infrastructure*,* and limited training opportunities […] (IDI-7).*

The interviewees reported that health leaders at all levels should better understand the healthcare system. This includes knowledge of the health system, its unique features, policies, and the local context. However, health leaders who are not from health fields without such knowledge were assigned to different levels of the health system, which often leads to poor decision-making and management:

*[…] Health leaders in the health system are sometimes not from health fields*,* which makes it difficult for them to understand the context and unique features of the healthcare system […] (IDI-2).*

Most interviewees stated that appointing health system leaders, such as heads of zonal health departments and district health offices, is not based on merit (competence). The leaders were assigned based on political affiliation, personal connections, and nepotism. The respondents pointed out the health system lacks accountability at both macro and micro levels. Political affiliations and personal connections seem to be prioritized over merit when appointing health leaders:

*[…] The appointment of health leaders at zones and district levels*,* including human resource managers*,* is not based on merit [competence] but on political affiliation*,* trust*,* networks*,* family*,* and relatives of political leaders. I was surprised to hear that district health office heads have been appointed based on the kebele they come from […] (KII-5).*

Health professionals need to maintain ethical and professional standards in their practice. However, most interviewees reported that health professionals often engaged in unethical behaviors, including insults, yelling, disrespect, disregard, abuse, refusal to carry out responsibilities, corruption, and fraud due to weak regulations and low awareness about health ethics:

*[…] In our [Amhara] culture*,* it is a norm for the community to come together and support a person who is ill by visiting them in his/her home and helping them recover without any expectation of payment…However*,* it is unfortunate that most health professionals working in health centers and primary hospitals do not give care with dignity and respect*,* which is against our culture […] (KII-3).*

Some interviewees also stated that it is common for health professionals working in public health facilities to intentionally engage in activities that could increase their profit in their private clinic or pharmacy, such as stocking out of drugs, damaging medical diagnostics equipment and reagents, and other similar actions. Unfortunately, there is no plausible control mechanism in place to prevent this issue from occurring. All interviewees reported several barriers in health centers and primary hospitals, such as the non-conducive working and living environment, lack of performance-based performance appraisal (e.g., giving the same evaluation results regardless of their performance), feeling of hopelessness, lack of training opportunities, and educational materials for personal and professional development. Some interviewed health professionals reported that their morality and humanity are the only forces that keep them updating their competence, staying and serving the community in their health facilities:

*[…] We [health professionals] are not being heard. There are no regulations*,* proper performance appraisals*,* or promotions. The working and living environment is not conducive*,* and there are no training opportunities for professional development. The only thing [that is sustaining us serving the public] is our morality and humanity […] (IDI-4).*

Most interviewees pointed out that capacity building through in-service training is abused in the health system. It serves as a means of getting money through per diem, and only a few individuals with strong links to managers frequently take the in-service training, including taking the same training several times, despite others not receiving a single in-service training throughout their workplace. Unfortunately, the health professionals who took the training do not share the knowledge and skills gained. The respondents also noted that the value of in-service training for improving health professionals’ competence is not evaluated in the real world:

*[…] The attitude of health leaders and health professionals towards in-service training is not good. They welcome in-service training*,* whether need-based or not*,* for money rather than to fill their capacity gap. Even after they finish the training*,* they throw the training materials*,* such as training modules*,* in the training hall […] (KII-3).*

Most interviewees indicated that continuous professional development, including in-service training, is not internally driven and home-grown. Instead, donor-driven in-service training is provided, whether need-based or not. The capacity-building training is left to the development partners of the health sector. No budget has been allocated for CPD and in-service training in either health centers or primary hospitals. Once health professionals get a license after graduating, that is sufficient to stay in their professions regardless of the state of their competence in the current health system:

*[…] Development partners initiate and provide disease-specific in-service training without conducting a needs assessment*,* which results in minimal or no value for the competence of health professionals […] (KII-5).*

Almost half of the interviewees indicated that health professionals work in health facilities with an expired, forged, or without a license due to an insufficient and inadequate regulatory framework:

*[…] In our health system*,* there is neither proper payment [for health professionals] nor regulation*,* so you can do what you would like to do […] (IDI-2).*

## Discussion

The findings of this study were discussed by using the WHO global competence framework [1] to frame quality PHC and Johnson and colleagues’ framework [17] to organize factors influencing health professionals’ competence in the delivery of quality PHC. The understanding of quality primary health care (PHC) and the required competencies for providing it vary among different groups of interviewees. Health professionals emphasize curative services, managers prioritize disease prevention, and academics and NGOs advocate for integrative health services when defining quality PHC and assessing the competence of health professionals.

Although the WHO’s global competency for UHC [1] considers people-centeredness as one of the main dimensions of quality PHC, there were different views on people-centeredness. For example, a few interviewees argued that people-centeredness is not feasible in the Ethiopian context, including Amhara region, due to the low health literacy of people and a shortage of health professionals, which makes them busy incorporating people’s preferences. This might be because some health professionals still maintain a paternalistic approach to delivering health care [20].

Most interviewees agreed that quality primary healthcare should include effective communication, evidence-informed decision-making, teamwork, collaboration, evidence-based practice, and high-standard personal conduct. This aligns with the WHO global competence for Universal Health Coverage (UHC) [1]. This study identified several facilitators and barriers to the competence of health professionals, which were discussed using Johnson and colleagues’ framework [17].

### Facilitators to the competence of health professionals in the provision of quality primary health care

This study highlights several facilitators enhancing health professionals’ competence across various subthemes, as discussed below using Johnson and colleagues’ framework [17].

#### Facilitators related to students

In the current study, facilitators for students include a substantial value for health professions, high cutoff points for entering health fields, and adoption of new strategies to prevent cheating and grade inflation in universities, consistent with previous studies [17, 19, 24]. These measures ensure that committed and qualified students enter the health professions.

#### Facilitators related to infrastructure and management

This study identified several facilitators regarding infrastructure and management, including advanced educational technology such as augmented realities and virtual patients, licensure and exit exams, graduate admission tests, and competency-based education. Professional councils, health science education centers, accreditation processes, strategic plans for competency assessment, and responsible structures for implementation ensure that educational standards are met. These findings align with previous studies [17, 19, 24].

#### Facilitators related to clinical and community practice sites

Regarding clinical and community practice sites, this study identified facilitators such as the construction of university teaching hospitals and the presence of research field sites and community-based education sites in some academic institutions. These facilities enhance practical learning opportunities, allowing students to gain hands-on experience in real-world settings, consistent with previous research findings [1, 17, 19].

#### Facilitators related to curriculum

The current study revealed curriculum-related facilitators, such as the availability of health professionals’ education experts, global and specific competency frameworks, and the introduction of competency-based education. Efforts to develop standard competency frameworks and entrustable professional activities (EPAs) further enhance the effectiveness of health education programs. These findings align with the WHO’s global competency framework for UHC and previous studies [1, 19, 24].

#### Facilitators related to teachers and preceptors

This study identified facilitators related to teachers and preceptors, such as higher diploma education and training in pedagogy and andragogy, often supported by NGOs. Capacity building through master’s and PhD programs further enhances the quality of education. These findings are consistent with Johnson and colleagues’ framework [17].

#### Facilitators related to the health system

This study highlights the potential of the education-for-life model in enhancing health professionals’ competence. In Ethiopia’s health system, CPD is a policy priority at national and subnational levels, supported by a national CPD roadmap and to link CPD with licensing. NGOs, universities, colleges, and CPD centers collaborate on capacity building, with IT-facilitated e-CPD playing a significant role. The presence of a regulatory body and strong recognition of a primary healthcare-oriented system further facilitate the development of competent health professionals, consistent with Johnson and colleagues’ framework and the WHO global competency framework for UHC [1, 19, 38, 39].

### Barriers to the competence of health professionals in the provision of quality primary health care

This study revealed that the pre-service education quality in academic institutions is poor, and the health system is not conducive to updating and developing emergent competencies. We discussed the barriers based on components proposed by Johnson and colleagues’ framework: students, infrastructure and management, clinical/community practical sites, curriculum, instructors and preceptors, and health system factors.

#### Barriers related to students

This study identified several issues related to health students, including problems with student selection, lack of motivation, negligence, credentialism, cheating, poor pre-higher education quality, use of forged evidence for entry, and awarding graduation certificates without program attendance. These findings align with previous studies conducted in low-and middle-income countries (19, 40–42). These issues prevent students from developing the competence to provide quality primary health care after graduation.

#### Barriers related to infrastructure and management

This qualitative study identified several barriers to infrastructure and management in academic institutions, such as limited capacity to adapt to disasters, weak regulation and governance, and poor coordination between health and education systems. It also highlighted limited national and international collaboration in Ethiopian academic institutions. These findings align with previous studies conducted in Ethiopia and elsewhere (19, 38, 40, 41, 43, 44). These issues imply that academic institutions fail to produce graduates who can address and respond to the health needs of individuals and the population.

#### Barriers related to clinical and community practice sites

This study highlights several barriers to community and clinical practice sites, including the lack of teaching hospitals, political unrest, insecurity, and inadequate clinical practice sites with large class sizes. These factors limit students’ practical experience opportunities, resulting in health graduates often lacking the necessary competencies, consistent with Johnson and colleagues’ framework [17].

#### Barriers related to the curriculum

This study also identified critical issues with pre-service education curricula, including outdated, fragmented, non-competency-based, unstandardized content, a mismatch between competencies and healthcare practice, and a lack of nationally agreed-upon competency frameworks and EPAs. Additional concerns include limited emphasis on inter-professional education, absence of needs assessments in curriculum design, and lack of a legal framework to harmonize curricula. Curricula also fail to incorporate indigenous knowledge, focusing too much on theoretical knowledge over practical sessions and emphasizing specialized biomedical care rather than primary health care. These findings indicate that current curricula are inadequate for preparing competent health professionals, supporting previous evidence [1, 17, 19].

#### Barriers related to instructors and preceptors

This study identified several barriers faced by teachers and preceptors, including pedagogically and clinically incompetent instructors and preceptors, demotivated instructors and preceptors, dual practice (also known as moonlighting), and non-compliance with curricula. Additionally, it highlighted a lack of partnerships with national and international faculty members, as well as insufficient capacity-building for instructors. These findings substantiate the previous evidence reflected in Johnson and colleagues’ framework and the WHO global competency framework of UHC [1, 17].

#### Barriers related to the health system

Despite the importance of lifelong learning through the education-for-life model to maintain and update health professionals’ competence in line with evolving health needs [19], this study notes that lifelong learning is either non-existent or insufficient at primary health care facilities. This issue stems from the absence of an operational and monitoring framework, limited budget for CPD and IST, lack of a legal framework for linking CPD with re-licensing, and absence of educational resources and infrastructure, weak coordination between the health system and the education system. Additionally, there is a lack of learning forums like morning sessions in primary health care facilities. This finding is consistent with those reported from other low- and middle-income countries [1, 38, 39].

## Conclusions

This study explored the factors influencing the competence of health professionals in delivering quality primary health care in the Amhara region, Ethiopia, using an exploratory qualitative approach. Educational technology, advanced instructional and educational design, the education-for-life model, and the presence of universities, colleges, CPD centers, and NGOs working on health workforce capacity development in the region have facilitated health professionals’ competence in providing quality primary health care. However, higher education institutions are not fulfilling their mission of producing competent health professionals and discharging their social responsibility as expected. Moreover, the insufficient and haphazard implementation of IST and CPD has contributed to health professionals’ low competence in providing quality primary healthcare. On the other hand.

### Implications for policy and practice

Based on the study’s findings, several policy and practice implications can enhance the competence of health professionals in Amhara region. Strengthening pre-service education is crucial through the implementation of fit-for-purpose recruitment standards, revising curricula to align with actual healthcare practice, and enhancing the recruitment and training of competent instructors, alongside a supportive educational policy and environment. Improving in-service training (IST) and continuing professional development (CPD) is essential, requiring sufficient budget allocation, increased availability of training opportunities, and provision of online courses and workshops. Enhancing the working and living conditions of health professionals is another key area that involves investing in infrastructure and providing incentives, such as housing, transportation, and allowances, to attract and retain professionals in rural and underserved areas. Strengthening the regulatory framework through strict enforcement of standards and better collaboration between the health system and higher education institutions is also essential. Ultimately, emphasizing competency-based education through hands-on training and practical experiences, as well as establishing mentorship programs, can bridge the gap between theory and practice.

### Limitations of the study and future research

Despite applying Guba and Lincoln’s trustworthiness criteria—credibility, transferability, dependability, and confirmability—this study may still be influenced by social desirability bias and subjectivity, as interviewees may tailor their responses to perceived expectations. Future research should incorporate multiple-choice questions and practical examinations based on the WHO global competency framework and Johnson et al.‘s framework to objectively assess the competence of health professionals and the factors influencing competence in delivering quality primary health care (PHC).

## Supplementary Information

Supplementary Material 1.

## Data Availability

Data is provided within the manuscript or supplementary information files.

## Abbreviations

CBE: Competency-Based Education
CPD: Continuous Professional Development
FMoH: Federal Ministry of Health
IST: In-service Training
MCC: Motivated, competent and compassionate
PHC: Primary Health Care
SPSS: Statistical Package for Social Science
UHC: Universal Health Coverage
WHO: World Health Organization
