# Supplementary material for: Facilitators and barriers to health professionals’ competence in delivering quality primary health care in the digital era in Amhara region, Ethiopia: an exploratory qualitative study
Source: BMC Prim Care. 2025 Sep 29;26:296. doi: 10.1186/s12875-025-03003-9 (PMC12482203; doi:10.1186/s12875-025-03003-9)
Supplement: Supplementary file 1 — Supplementary Material 1. [file 12875_2025_3003_MOESM1_ESM.docx]

**Interview guides**

**I. Key informant interview guide (for academia, health managers, and NGO participants)**

1. In your opinion, how do you describe health professionals’ competence in providing quality primary healthcare (probe on competence in people-centeredness, decision making, effective communication, evidence-based practice, collaboration including teamwork, personal conducts)?
2. In your perspective, how do you describe barriers to quality pre-service education of health professionals to provide quality primary health care (probe on students’ attributes, instructors/preceptors’ attributes, clinical/community practical sites, infrastructure and management, curricula being implemented, regulation and quality assurance, contextual factors, crisis including COVID-19 and conflict).
3. In your view, how do you describe the facilitators of quality pre-service education of health professionals(probe on students’ attributes, instructors/preceptors’ attributes, clinical/community practical sites, infrastructure and management, curricula being implemented, regulation and quality assurance, contextual factors, crisis including COVID-19 and conflict)
4. In your view, how do you describe barriers of CPD and in-service training of health professionals to provide quality primary health care (probe on financial barriers, regulation/governance, attributes of health professionals, attributes of health system and leaders, contextual factors and crisis including COVID-19 and conflict).
5. In your view, how do you describe the facilitators of CPD and in-service training of health professionals to provide quality primary health care?
6. What recommendations do you have to improve pre-service and in-service health professionals’ education, including implementing the education for life model?
7. Do you want to add anything about the barriers and facilitators of health professionals’ competence?

**II. In-depth interview guide (for health professionals)**

1. How do you describe your life experience and your competence to provide quality primary health care (probe on competence in people-centeredness, decision-making, effective communication, evidence-based practice, collaboration including teamwork, and personal conduct)?
2. Would you explore the barriers to quality pre-service education of health professionals to provide quality primary health care (probe on students’ attributes, instructors/preceptors’ attributes, clinical/community practical sites, infrastructure and management, curricula being implemented, regulation and quality assurance, contextual factors, crisis including COVID-19 and conflict)?
3. Would you explore the facilitators of quality pre-service education of health professionals(probe on students’ attributes, instructors/preceptors’ attributes, clinical/community practical sites, infrastructure and management, curricula being implemented, regulation and quality assurance, contextual factors, crisis including COVID-19 and conflict)
4. Would you explore the barriers of CPD and in-service training of health professionals to provide quality primary health care (probe on financial barriers, regulation/governance, attributes of health professionals, attributes of the health system and leaders, contextual factors and crisis including COVID-19 and conflict)?
5. Would you explore the facilitators of CPD and in-service training of health professionals to provide quality primary health care).
6. What recommendations do you have to improve pre-service and in-service health professionals’ education, including implementing the education for life model?
7. Do you want to add anything about the barriers and facilitators of health professionals’ competence?
